# The impact of an hematocrit of 20% during normothermic cardiopulmonary bypass for elective low risk coronary artery bypass graft surgery on oxygen delivery and clinical outcome – a randomized controlled study [ISRCTN35655335]

**DOI:** 10.1186/cc4891

**Published:** 2006-04-10

**Authors:** Christian von Heymann, Michael Sander, Achim Foer, Anja Heinemann, Bruce Spiess, Jan Braun, Michael Krämer, Joachim Grosse, Pascal Dohmen, Simon Dushe, Jürgen Halle, Wolfgang F Konertz, Klaus-Dieter Wernecke, Claudia Spies

**Affiliations:** 1 Department of Anesthesiology and Intensive Care Medicine, Charité – University Hospital Berlin, Charité Campus Mitte, Berlin, Germany; 2 Department of Anesthesiology and the Virginia Commonwealth University Reanimation Engineering Shock Center (VCURES), Virginia Commonwealth University Medical Center, Richmond, Virginia, USA; 3 Department of Cardiovascular Surgery, Charité – University Hospital Berlin, Charité Campus Mitte, Berlin, Germany

## Abstract

**Introduction:**

Cardiopulmonary bypass (CPB) induces hemodilutional anemia, which frequently requires the transfusion of blood products. The objective of this study was to evaluate oxygen delivery and consumption and clinical outcome in low risk patients who were allocated to an hematocrit (Hct) of 20% versus 25% during normothermic CPB for elective coronary artery bypass graft (CABG) surgery.

**Methods:**

This study was a prospective, randomized and controlled trial. Patients were subjected to normothermic CPB (35 to 36°C) and were observed until discharge from the intensive care unit (ICU). Outcome measures were calculated whole body oxygen delivery, oxygen consumption and clinical outcome. A nonparametric multivariate analysis of variance for repeated measurements and small sample sizes was performed.

**Results:**

In a total of 54 patients (25% Hct, *n *= 28; 20% Hct, *n *= 26), calculated oxygen delivery (*p *= 0.11), oxygen consumption (*p *= 0.06) and blood lactate (*p *= 0.60) were not significantly different between groups. Clinical outcomes were not different between groups.

**Conclusion:**

These data indicate that an Hct of 20% during normothermic CPB maintained calculated whole body oxygen delivery above a critical level after elective CABG surgery in low risk patients. The question of whether a transfusion trigger in excess of 20% Hct during normothermic CPB is still supported requires a larger prospective and randomized trial.

## Introduction

Hemodilution occurs during cardiac surgery when cardiopulmonary bypass (CPB) is instituted. Hemodilution reduces blood viscosity and vascular resistance, and may increase large vessel blood flow to maintain whole body oxygen delivery [1]. It does appear that the microcirculation can regulate red cell flow and concentration over a wide range of hematocrit (Hct) levels [2]. Of interest, a 43% increase in cerebral blood flow has been described for a 31% reduction in hemoglobin concentration during CPB [3].

Debate exists on the minimum safe level Hct necessary to maintain oxygen delivery (DO_2_) during CPB. Hct level is used as a measure for triggering transfusion, but transfusion carries a wide range of complications and appears to worsen outcome after coronary artery bypass graft (CABG) surgery [4]. Moreover, it was shown that aged red blood cells from the blood bank delivered less oxygen to tissue than fresh blood [5] and transfusion of allogeneic red blood cells was ineffective in improving skeletal muscle oxygen tension after CABG surgery compared to ventilation with 100% oxygen [6]. Results from the literature do demonstrate a relationship between low Hct and adverse outcomes. Fang and colleagues [7] found a doubling (2.7-fold) of mortality when the lowest nadir Hct reached 14% during CPB. Later, Defoe and co-workers [8] reported that an Hct <19% during CPB was associated with a two-fold increase in hospital mortality and a trend towards increased risk of death for an HCT <23%. Neither Fang and colleagues nor Defoe and colleagues investigated transfusion as a covariate or confounder. It could well be that these data were driven by transfusion risks and not critical Hct and oxygen delivery. In a recent study, Habib and colleagues [9] described a sigmoidal association between the extent of hemodilutional anemia and acute renal failure after cardiac surgery. The relationship between nadir hematocrit and acute renal failure was worsened by intra-operative transfusions in this study. The authors concluded that hemodilutional anemia did not necessarily cause but increased the likelihood of acute renal failure. Furthermore, transfusion of packed red blood cells may not be the appropriate treatment for renal injury. This study supports the hypothesis that transfusion may be a risk factor for acute renal failure after CPB.

Most data on the relationship between hemodilutional anemia and outcome have been measured during hypothermic (28 to 32°C), moderate hypothermic (32 to 34°C) or normothermic CPB. Temperature regulation favoring hypothermia during CPB was found to reduce cerebral oxygen consumption [10], preserve myocardial function [11] and to reduce whole body oxygen metabolism [12]. However, normothermia for CPB has emerged as an alternative technique for temperature regulation during CPB [13] that has been associated with a comparable clinical outcome [14,15]. Hemodilution to an Hct of 0.10 (± 0.02) has been shown to increase cerebral blood flow compared to baseline to a greater extent at 38°C than at 28°C, and at 28°C than at 18°C [16]. Cerebral metabolic rate (CMRO_2_) was kept stable at Hct levels of 0.14, 0.11 and 0.10 in the respective temperature groups. The authors of this animal study concluded that the compensatory increase in cerebral blood flow at an Hct of 0.14 met the increased rate of cerebral oxygen demand even at a body temperature of 38°C. Although results from controlled clinical studies reported similar or beneficial outcome after normothermic CPB [17,18], cardiac surgical practice still performs CPB over a broad range of temperatures.

Results from controlled trials investigating the effect of hemodilution on oxygen delivery and clinical outcome in patients undergoing normothermic CPB have not yet been published. Therefore, the objective of this prospective, randomized and controlled study was to investigate oxygen delivery and consumption and the clinical outcome of patients who were randomly allocated to one of two Hcts (20% or 25%) during normothermic CPB.

## Materials and methods

### Group assignment

After institutional approval by the local ethics committee and preoperative written informed consent, 57 patients were considered eligible for this randomized, controlled clinical trial from February 2004 until November 2004. Patients were allocated to the trial groups according to a computer-generated random list. One patient had to be excluded from analysis as the donated autologous blood showed multiple clots and could not be retransfused. Informed consent was withdrawn by one patient and one patient had to be excluded due to the preoperative decision for a combined surgical procedure. In total, 54 patients (28 in the 25% Hct group and 26 in the 20% Hct group) remained for statistical analysis according to the Full Analysis Set (Intention To Treat).

Inclusion criteria were age >18 and <75 years, elective coronary artery bypass graft surgery, weight >70 kg and preoperative Hct >36% (hemoglobin >12 g/dl).

Exclusion criteria were withdrawal of consent, Jehova's Witnesses, stroke in patient's history or with persistent neurological residue, unilateral occlusion of carotid artery >70% or bilateral occlusion of carotid artery >50%, combined cardiac procedure, left ventricular ejection fraction <40%, unstable angina, a left main stem stenosis >70%, ventricular arrhythmia >LOWN IVa, symptomatic chronic pulmonary disease requiring long-term medication or FEV_1 _<70% or FEV_1_/VC max <70% or partial pressure of oxygen in arterial blood (paO_2_) <60 mmHg, known acute or chronic hepatitis or hepatic disease with impaired synthesis of coagulation factors or bilirubin >2.0 mg/dl, known inflammatory bowel disease, known renal insufficiency or anuric renal failure or creatinine >1.5 mg/dl, ingestion of aspirin or clopidogrel until 3 days prior to surgery, and treatment with glycoprotein-receptor antagonists within 2 days before surgery. Furthermore, patients were excluded from the study if the Hct could not maintained within the targeted range during CPB, if an emergency situation (for example, cardiopulmonary resuscitation, acute right or left ventricular failure) occurred before initiation of CPB and if the autologous blood could not be retransfused.

### Anesthetic and CPB technique, isovolemic hemodilution and management in the intensive care unit

The standard anesthetic practice was an opioid-based anesthetic supplemented with midazolam and isoflurane as required. In all patients, a femoral artery was cannulated with a 4-Fr.-cannula (Pulsiocath, Pulsion, Munich, Germany) prior to induction of anesthesia. A central venous catheter and a pulmonary artery catheter (Thermodilution Catheter, Arrow, Reading, PA, USA) were inserted via the right internal jugular vein.

The standardized CPB priming consisted of 600 ml of crystalloid fluid, 500 ml of 10% hydroxyethylstarch solution and a total dose of 50,000 KIU aprotinin per kg bodyweight prior to and during CPB. Pump flow was adjusted to maintain a mean arterial pressure (MAP) of 55 to 60 mmHg and an oxygen saturation >75% during CPB. Norepinephrine was used during CPB when MAP could not be maintained within the targeted range by adjusting the pump flow. During bypass an arterial partial pressure of oxygen of 150 to 250 mmHg was maintained. Body temperature was kept between 35.5 and 36°C during CPB. CPB technique was normothermic using intermittent antegrade warm blood cardioplegia as described by Calafiore and colleagues [19]. Before institution of CPB, isovolemic hemodilution using a hydroxyethylstarch solution 130/0.4 (Voluven^®^, Fresenius-Kabi, Bad Homburg, Germany) was performed to reduce the Hct to a level of 5 ± 1% above the target Hct level of 20 ± 1% or 25 ± 1%. For all measurements of Hct and blood lactate, a blood gas analyzer (ABL-700 series, Radiometer, Copenhagen, Denmark) was used.

Measurements of Hct were done 2 and 5 minutes after initiation of CPB and every 15 minutes when the target hematocrit was stable. In cases where the initial Hct exceeded the targeted Hct range, crystalloid fluid was substituted, and if the hematocrit was below the target range, autologous blood was transfused.

In the intensive care unit (ICU), patients were extubated as soon as possible after an observation period of 6 hours if they fulfilled the following criteria: paO_2 _>60 mmHg with an inspired oxygen fraction (FiO_2_) of 40%, adequate neurological reaction and sufficient muscle strength. Packed red blood cells were substituted according to the following protocol: Hct <23% or lactic acidosis or ST-segment elevation or secondary organ failure attributed to hemodilutional anemia. In the ICU, the patients were not infused with crystalloids according to a standard protocol. Hydroxyethylstarch solution (6% HES 130/0.4) was given when patients showed clinical symptoms of hypovolemia.

Discharge from ICU to the intermediate care unit was feasible when patients were in a stable clinical condition, that is, awake without neurological deficit or agitation, = 5 μg/kg/minute dopamine or no inotrope support, paO_2 _>60 mmHg with an oxygen insufflation of 4 l per minute and a normal partial pressure of carbon dioxide in arterial blood (paCO_2_) and no need for continuous loop diuretics to maintain urinary output or renal replacement therapy.

### Outcome measures

Primary outcome measures of this trial were calculated whole body oxygen delivery, oxygen consumption and mixed venous blood lactate during CPB, at the end of surgery and in the ICU (one, six and 18 hours after admission). Secondary outcome measures were: drainage loss, transfusion utilization and Hct in the ICU, incidence of secondary organ failure, hemodynamic parameters and stay in ICU.

Oxygen delivery and oxygen consumption were calculated using standard formulae (see Additional file 1). Hemodynamic parameters such as MAP, central venous pressure, mean pulmonary artery pressure and pulmonary artery occlusion pressure were measured before hemodilution, at the end of surgery after retransfusion of autologous blood, one and six hours after admission to the ICU and before discharge from the ICU. Cardiac index, systemic vascular resistance and pulmonary vascular resistance were calculated using standard formulas whereas extravascular lung water and intrathoracic blood volume index were calculated at the same time points using the PiCCO plus-monitor (Pulsion, Munich, Germany). During CPB, pump-driven cardiac index, body temperature and blood lactate were recorded 15 minutes after institution and at the end of CPB whereas the cumulative amount of norepinephrine and urine volume were taken at the end of CPB. The incidence of acute cardiac failure, defined as need for epinephrine and/or enoximone, the need for intraaortic balloon counterpulsation for separation from CPB, and the dosage of dopamine for weaning from CPB were recorded.

In the ICU, the following indicators for secondary organ failure were assessed: neurological complications defined as transitory ischemic attack, agitated arousal reaction or palsy of extremities or hemiplegia. Myocardial infarction was determined by electrocardiogram (new Q-wave, ST-elevations >2 mm) and a ratio of creatine kinase and myocardial subtype of creatine kinase >10%. Acute cardiac failure was defined as the need for inotrope support (epinephrine, norepinephrine or phosphodiesterase inhibitors), respiratory failure as the need for reintubation due to respiratory failure, prolonged respiratory support (>24 hours) or the need for continuous positive airway pressure breathing. Renal insufficiency was assumed when patients required renal replacement therapy, continuous intravenous loop diuretics or increase of creatinine >2.0 mg/dl. Additionally, chest drainage loss, transfusion requirements and Hct, cumulative urine volume, creatinine, ICU stay in hours and mortality were recorded.

### Statistical methods

Because of the limited sample sizes and/or non-symmetrically distributed observations we applied only nonparametric statistics. Results were expressed as median and interquartile range in the case of continuous variables. Absolute and relative frequencies were used for categorical and dichotomous variables. The effect of hemodilution regarding primary and secondary outcomes was analyzed using χ^2 ^or Fisher's exact test for categorical and dichotomous variables, respectively. In the case of continuous variables, we applied the Mann-Whitney *U *test for inter-group analysis. A non-parametric multivariate analysis of variance (nonparametric MANOVA) for repeated measurements and small sample sizes in two and three-factorial designs [20], respectively, was performed in order to take the whole time courses into consideration simultaneously. Multiple tests for differences between the groups in question have been regarded as exploratory ones and were not adjusted for multiplicity. A two-tailed *p *< 0.05 was considered statistically significant. The statistical analysis was performed using SPSS for Windows, 11.0 (SPSS, Inc., Chicago, IL, USA).

## Results

Basic patient characteristics (Table 1) did not differ between the groups. The majority of patients were men (*n *= 52) with two women who were randomized in the higher hematocrit group.

Figure 1 gives the results for oxygen delivery and consumption throughout the study period. Calculated oxygen delivery differences between groups almost reached statistical significance at the end of surgery (767 versus 647 ml/m^2^/minute, *p *= 0.07). Oxygen consumption was significantly lower in the 20% Hct group 6 hours after admission to the ICU (299 versus 246 ml/m^2^/minute, *p *= 0.05). In the non-parametric MANOVA the difference in oxygen delivery did not remain significant (*p *= 0.11) between groups over the study period whereas oxygen consumption tended to be lower (*p *= 0.06) in the 20% Hct group. The blood lactate levels were not different between groups throughout the study period. For blood lactate, the non-parametric MANOVA did not find a significant difference between groups throughout the study period (*p *= 0.6).

The Hct was significantly lower in the 20% Hct group from the end of surgery until the end of ICU stay. Notably, an Hct of 30% was reached in the 20% Hct group at 6 hours after admission to the ICU (30.0 versus 33.1%, *p *< 0.01). The non-parametric MANOVA confirmed the significantly lower Hct levels in the 20% Hct group (*p *< 0.01).

Table 2 shows intra-operative results (hemodynamic measurements, dosage of norepinephrine, urine volume during CPB) that were measured prior to, during and after CPB. All intra-operative measures were not significantly different between groups.

The clinical outcome in the ICU with regard to chest drainage loss, transfusion use and secondary organ failure are given in Table 3. Blood loss from chest drainage (*p *= 0.28), the incidence of neurological complications (*p *= 0.99), cardiac (*p *= 0.99), respiratory (*p *= 0.99) and renal failure (*p *= 0.99) and the combined endpoint of organ failure (*p *= 0.57) were not different between the groups. Five patients in the 20% Hct group and one patient in the 25% Hct group (*p *= 0.10) were transfused in the ICU. In particular, no acute myocardial infarction was recorded. ICU stay in hours was not significantly different between the groups.

One patient in the 20% Hct group died of septic multi-organ failure due to pneumonia occurring on postoperative day 3, accounting for the overall mortality of 1.9% (Table 3).

## Discussion

### Oxygen delivery, oxygen consumption and blood lactate

This study demonstrated that an Hct of 20% during normothermic CPB for elective CABG surgery did not 'critically' reduce calculated whole body oxygen delivery. Over the study period, calculated oxygen delivery was maintained above a critical threshold of 330 ml/minute/m^2 ^in anesthetized humans, as reported by Shibutani and colleagues [21]. An increase in cardiac index in the 20% Hct group (Table 4) may have compensated for the reduction in oxygen carrying capacity and prevented DO_2 _from being significantly reduced. It may also be that the microcirculation compensated for the differences in Hct levels used in our study [2]. Mathru and colleagues [22] reported results from 8 anesthetized patients who were exposed to an Hct of 15% after elective CABG surgery and a body temperature of 37°C, which led to a significant, but not 'critical', reduction in DO_2_. Patients in the 20% Hct group used significantly less oxygen in the ICU (at six hours after admission; Figure 1). In both groups, oxygen consumption continuously increased from the lowest values at the end of surgery to the highest values before discharge from the ICU while oxygen delivery was maintained well above critical levels. Forces upon oxygen consumption during the ICU period and after extubation, which include, for example, lower level of sedation, increased muscular activity, fever or pain, may have caused the increase in oxygen consumption. Prior work in awake humans has shown that oxygen consumption is stable until an Hct of 15% [23], indicating that hemodilution alone may not change oxygen consumption. Since the oxygen extraction rate also increased over the study period in both groups (Figure 1), a higher whole body oxygen demand has to be assumed. A similar increase in oxygen consumption and oxygen extraction rate was reported to occur in general surgery [24] and cardiac surgical patients [25] and seems to represent a hypermetabolic response to the surgical trauma rather than to CPB [25].

Both groups had normal blood lactate levels throughout the study, indicating that a gross mismatch of whole body oxygen delivery and consumption during CPB did not occur. This further supports our conclusion that hemodilution to 20% Hct maintained DO_2 _above a critical level and may be considered safe in regard to adequate whole body oxygen supply in normothermic CPB for elective CABG surgery in low risk patients.

### Clinical outcome

The clinical outcome in regard to drainage loss, transfusion requirements and secondary organ failure were not different between the study groups. In particular, no myocardial infarction was detected in our group of patients, which may support the hypothesis that myocardial oxygen delivery during and after CPB was maintained in the 20% Hct group. Our results are in accordance with previous results that an Hct of <34% on admission to the ICU (25.8% versus 23.5% in our study groups, respectively was not associated with a significantly higher rate of myocardial ischemia [26].

Moreover, an Hct as low as 20% during normothermic CPB was not associated with a higher incidence of acute renal failure (2/54 (3.7%) patients, 1 patient in each group), which confirms previous results [27]. It has to be assumed that oxygen delivery to the kidneys was maintained since postoperative creatinine levels, urine volumes and usage of loop diuretics (data not shown) were not significantly different between groups. This may suggest that an Hct of 20% during CPB may preserve renal function in patients without preoperative renal impairment. These results are in contrast to recent studies [28,29] reporting an association between low Hct during CPB and acute renal failure, although these studies were not able to show a cause-effect relationship for low Hct and acute renal failure.

The median stay in the ICU in our patients was 22 hours in the 25% Hct group versus 23 hours in the 20% Hct group (*p *= 0.24) and confirms previous results from elective CABG surgery [30]. The combined endpoint of postoperative organ failure (neurological, cardiac, respiratory and renal) was not significantly different between groups and may have contributed to this outcome. This may indicate that an Hct of 20% during normothermic CPB in a group of low-risk CABG patients does not necessarily impair clinical outcome.

### Study limitations

Our study does have some limitations. First, the sample size of this pilot study was generally not large enough to detect the differences observed with sufficient power. Therefore, our results may serve as a database for larger studies investigating the effect of hemodilution during CPB. A power calculation for the outcome 'ICU stay' with the given sample sizes would correspond to a power of not more than 20% for 'ICU stay' whereas for 'oxygen delivery (DO_2_)' would result in a sufficient power of more than 80%. A sufficiently powered clinical trial investigating 'mortality' as a clinical endpoint would require a far larger number of patients to detect a clinically relevant difference between the groups.

Second, patients studied were low risk patients. This precludes that our results can be generalized to cardiac patients with certain comorbidities in whom an individual risk-benefit analysis prior to transfusion is required. A univariate analysis of all risk factors that may have influenced the length of ICU stay was performed but did not find the lowest Hct during CPB to be predictive for ICU stay. Therefore, and due to the limited sample size, a multivariate analysis was not considered appropriate. Third, there were only two women in our study population, which does not reflect the gender distribution of CABG patients. In particular, women with a lower body weight are at risk of being exposed to a low Hct and allogeneic blood products during CPB. Therefore, our results need to be confirmed in a larger study including a sufficient number of women. Fourth, five times as many patients in the 20% Hct group were transfused after surgery. This was not statistically significant (*p *= 0.1) but may have become so in a larger study. Transfusions in the postoperative period may have non-beneficial effects on safety and outcome in cardiac surgery [3,4] and need to be as closely controlled as possible during CPB. Using a more restrictive transfusion threshold as suggested by Paone and colleagues [31], who transfused cardiac surgical patients in the ICU at an Hct of less than 20% without observing any adverse effects on outcome. This may have prevented some patients from being transfused in our study.

## Conclusion

Our results from a small and low risk group of CABG patients suggest that hemodilution to an Hct of 20% during normothermic CPB did not induce a 'critical' imbalance between oxygen delivery and consumption that would have resulted in a worse clinical outcome. Although these results require confirmation by larger studies, patients without severe comorbidities who are at a high risk of receiving allogeneic blood products may safely tolerate an Hct of 20% during normothermic CPB. If further investigations determine an Hct of 20% during normothermic CPB to be safe, a reduction in exposure to allogeneic blood products seems to be possible.

## Key messages

• 	An Hct of 20% versus 25% during normothermic CPB for CABG surgery did not impair oxygen delivery.

• 	No significant difference in oxygen consumption between the study groups was detected, indicating that a critical 'oxygen balance' was not induced by lowering the hematocrit to 20% during normothermic CPB.

• 	The clinical outcome of the patients was not significantly different between the groups, although the study was not sufficiently powered for this outcome.

## Abbreviations

CABG = coronary artery bypass graft; CPB = cardiopulmonary bypass; DO_2 _= oxygen delivery; Hct = hematocrit; ICU = intensive care unit; MAP = mean arterial pressure; paO_2 _= partial pressure of oxygen in arterial blood.

## Competing interests

The authors declare that they have no competing interests.

## Authors' contributions

CvH: conception and design of the work, sampling of data, analysis of the data and drafting the article for content. MS: conception and design of the work, sampling of data, analysis and interpretation of the data and revising the article for content. AF: conception and design of the work, interpretation of the data and revising the article for content. BDS: interpretation of the data, revising the article for content and approval of the version to be published. AH: conception and design of the work and sampling of data. JB: conception and design of the work, interpretation of the data and revising the article for content. MK: conception and design of the work, sampling of data, revising the article for content. JG: conception and design of the work, sampling of data, interpretation of the data and revising the article for content. PD: conception and design of the work, interpretation of the data and revising the article for content. SD: conception and design of the work, interpretation of the data and revising the article for content. JH: conception and design of the work, sampling of data and revising the article for content. WFK: revising the article for content and approval of the version to be published. K-DW: analysis and interpretation of the data, revising the article for content, approval of the version to be published. CS: conception of the work, interpretation of the data, revising the article for content and approval of the version to be published.

## Supplementary Material

Additional file 1 Standard formulae used for calculating outcome measures. Click here for file
