# Supplementary material for: The impact of an hematocrit of 20% during normothermic cardiopulmonary bypass for elective low risk coronary artery bypass graft surgery on oxygen delivery and clinical outcome – a randomized controlled study [ISRCTN35655335]
Source: Crit Care. 2006 Apr 10;10(2):R58. doi: 10.1186/cc4891 (PMC1550910; doi:10.1186/cc4891)
Supplement: Additional file 1 — Standard formulae used for calculating outcome measures. [file cc4891-S1.doc]

Appendix:

Cardiac index (l x min^-1^ x m^-2^)

CI = CO/ m^-2^

CI denotes cardiac index and CO denotes cardiac output

Oxygen delivery (mL x min^-1^ x m^-2^)

DO_2_ = CaO_2_ x CI x 10

DO_2_ denotes oxygen delivery, CaO_2_ denotes arterial oxygen content, and CI denotes cardiac index

Oxygen consumption (mL x min^-1^ x m^-2^)

VO_2_ = (CaO_2_ – CvO_2_) x CI x 10

VO_2_ denotes oxygen consumption, CaO_2_ denotes arterial oxygen content, CvO_2_ denotes oxygen content, and CI denotes cardiac index

Arterial oxygen content (mL/dL)

CaO_2_ = (Hb x 1.39 x SaO_2_) + (0.0031 x paO_2_)

CaO_2_ denotes arterial oxygen content, Hb denotes haemoglobin concentration, SaO_2_ denotes arterial oxygen saturation, and paO_2_ denotes arterial partial pressure of oxygen: 1.39 is the oxygen-carrying capacity of hemoglobin (mL O_2_/ gram Hb); 0.0031 is the solubility coefficient of oxygen in plasma (mL O_2_/mmHg pO_2_)

Mixed venous oxygen content (mL/dL)

CvO_2_ = (Hb x 1.39 x SvO_2_) + (0.0031 x pvO_2_)

CvO_2_ denotes mixed venous oxygen content, Hb denotes hemoglobin concentration, SvO_2_ denotes mixed venous oxygen saturation, and pvO_2_ denotes mixed venous partial pressure of oxygen.

Systemic vascular resistance (dyne x s x cm^-5^)

(MAP – CVP) x 79.96

MAP denotes mean arterial pressure and CVP denotes central venous pressure.

Pulmonary resistance (dyne x s x cm^-5^)

(MPAP – PAOP) x 79.96

MPAP denotes mean mean pulmonary artery pressure and PAOP denotes pulmonary artery occlusion pressure.
